# Autophagy in Inflammatory Diseases

**DOI:** 10.1155/2011/732798

**Published:** 2011-11-30

**Authors:** Alexander J. S. Choi, Stefan W. Ryter

**Affiliations:** ^1^College of Arts and Sciences, Boston College, 140 Commonwealth Avenue, Chestnut Hill, MA 02467, USA; ^2^Pulmonary and Critical Care Medicine, Brigham and Women's Hospital, Harvard Medical School, 75 Francis Street, Boston, MA 02115, USA; ^3^Adjunct Scientist, Lovelace Respiratory Research Institute, Albuquerque, NM 87108, USA

## Abstract

Autophagy provides a mechanism for the turnover of cellular organelles and proteins through a lysosome-dependent degradation pathway. During starvation, autophagy exerts a homeostatic function that promotes cell survival by recycling metabolic precursors. Additionally, autophagy can interact with other vital processes such as programmed cell death, inflammation, and adaptive immune mechanisms, and thereby potentially influence disease pathogenesis. Macrophages deficient in autophagic proteins display enhanced caspase-1-dependent proinflammatory cytokine production and the activation of the inflammasome. Autophagy provides a functional role in infectious diseases and sepsis by promoting intracellular bacterial clearance. Mutations in autophagy-related genes, leading to loss of autophagic function, have been implicated in the pathogenesis of Crohn's disease. Furthermore, autophagy-dependent mechanisms have been proposed in the pathogenesis of several pulmonary diseases that involve inflammation, including cystic fibrosis and pulmonary hypertension. Strategies aimed at modulating autophagy may lead to therapeutic interventions for diseases associated with inflammation.

## 1. Introduction

### 1.1. Inflammation

Acute inflammation acts as part of the host's innate protective response to infection or tissue injury. Endothelial cell injury or microbial infection causes changes in vascular permeability, local edema, and in the distribution of chemoattractants [1, 2]. The activation of endothelial cells allows the transmigration of leukocytes, initially primarily neutrophils (polymorphonuclear (PMN) cells), to the site of tissue injury [3]. Finally, macrophages uptake apoptotic PMN cells, cellular debris, and invasive pathogens *via* phagocytosis during the resolution of acute inflammation, which leads to neutrophil clearance and the release of anti-inflammatory cytokines such as transforming growth factor-*β*1. The resolution program ends with the efflux of macrophages from the site of inflammation through lymphatics [4]. However, aberrant inflammatory responses can be associated with a wide range of acute, chronic, and systemic inflammatory disorders, such as cardiovascular disease, asthma, inflammatory bowel disease, rheumatoid arthritis [1], and cystic fibrosis [5].

In recent years, emerging evidence has indicated that the process of macroautophagy may play an essential role for the host during bacterial clearance [6] as well as interact with inflammatory processes, and thereby potentially impact the outcome of disease progression. 

### 1.2. Autophagy

Macroautophagy (autophagy) refers to the cellular pathway for the degradation or disposal of organelles and proteins *via* lysosomal processes. The autophagy mechanism involves double-membrane vesicles, called autophagosomes or autophagic vacuoles (AVs) that target and engulf cytosolic material, which may include damaged organelles or denatured proteins. The autophagosomes fuse with lysosomes to form single-membrane autolysosomes. Lysosomal enzymes facilitate this degradation process to regenerate metabolic precursor molecules (i.e., amino acids and fatty acids), which can be used for anabolic pathways and energy production [7–12]. This process may thereby prolong cellular survival during starvation. During infection, autophagy assists in the immune response by providing a mechanism for the intracellular degradation of invading pathogens such as bacteria [5]. Furthermore, autophagy influences the immune system during pathogen clearance by regulating antigen presentation, lymphocyte development, and proinflammatory cytokine production [13]. However, the mechanism for the involvement of autophagy in cytokine secretion remains poorly understood. In addition to macroautophagy, several other subtypes of autophagy exist, including microautophagy and chaperone-mediated autophagy [14].

At least 30 autophagy-related (Atg) genes have been determined, primarily in yeast. The homologues of many of these Atg genes have been shown to participate in the regulation of autophagy [14–16]. Among these, Beclin 1 (the mammalian homolog of yeast Atg6) represents a major autophagic regulator and tumor suppressor protein [17]. 

Recent studies suggest that autophagy evolved as a homeostatic response for unicellular eukaryotic organisms. Moreover, the same autophagy process could be used for diverse functions in more complex multicellular organisms in response to various stressful stimuli [5]. Therefore, the evolving understanding of autophagy and its interaction with other intracellular processes may reshape our knowledge and lead to the development of therapies for inflammatory disorders.

Recent studies suggest that the process of autophagy may be more selective than originally described such that there exist specific molecular mechanisms that regulate the autophagy-dependent intracellular degradation of bacteria, denatured protein aggregates, mitochondria, and other subcellular substrates [18]. Autophagy plays an important role in the maintanance of healthy organelle populations by eliminating damaged specimens (e.g., mitochondria and endoplasmic reticulum (ER)). 

In addition to providing basic homeostatic functions, autophagy can potentially impact other vital cellular processes, including programmed cell death (i.e., apoptosis). The complex relationship between autophagy and cell death pathways has been reviewed elsewhere [19–21]. It is now well recognized that autophagy can exert a critical influence on systemic immune and inflammatory responses and on the specific cell types that mediate these responses. This paper will summarize how these dynamic relationships influence the pathogenesis of several diseases, including pulmonary and systemic diseases, where inflammatory processes have been implicated. 

### 1.3. Autophagic Machinery

The activation of the autophagic pathway involves the assembly of several macromolecular signaling complexes [14, 16]. These include the mammalian target of rapamycin (mTOR) complex-1 (mTORC1) which consists of mTOR and several accessory proteins. The mTORC1 regulates a macromolecular substrate complex (mTOR substrate complex) containing the mammalian uncoordinated-51-like protein kinase ULK1 (the mammalian homolog of yeast Atg1) and other factors (i.e., Atg13, FIP200, and Atg101) [22–25]. The mTOR pathway negatively regulates autophagy during nutrient-rich conditions. Through this pathway, starvation and stimulation with the immunosuppressive drug rapamycin potently induce autophagy, by inhibiting mTOR kinase activity, and thereby permitting the activation of ULK1 kinase, a major initiator of autophagy [26, 27]. 

The *de novo* formation of the autophagosome membrane, presumably originating from the ER, requires a major macromolecular regulatory complex that includes Beclin 1 and Vps34, a Class III phosphatidylinositol-3-kinase (PIK3C3) [28]. Several additional proteins can interact with and influence the activity of this complex (e.g., Rubicon, UVRAG, and Bcl-2 family proteins, etc.). Activation of PIK3C3 activity leads to the increased production of phosphatidylinositol-3-phosphate (PI-3P) which regulates the initial steps in autophagosome formation [28]. 

The subsequent elongation of autophagosomes requires the activation of two ubiquitin-like conjugation systems [14, 16, 30]. First, the ubiquitin-like protein Atg12 is conjugated to Atg5 by Atg7 (E1-like) and Atg10 (E2-like) enzymes. The Atg5–Atg12 complex in turn associates with Atg16L (the mammalian homolog of yeast Atg16). The resulting multimeric complex assists in the elongation of the autophagic membrane [30]. 

A second conjugation system requires the ubiquitin-like protein, microtubule-associated protein-1 light chain 3 (LC3), and the mammalian homologue of yeast Atg8 [31, 32]. Several homologues of LC3 (i.e., LC3B) and related cellular cognate proteins (i.e., GABARAP) are conjugated with the phospholipid phosphatidylethanolamine (PE) [32, 33]. Atg4B catalyzes the proteolytic processing the LC3 proform to generate the cleaved form LC3-1. Conjugation of LC3-I with PE is subsequently catalyzed by Atg7 (E1-like) and Atg3 (E2-like) activities [30]. In mammals, the conversion of LC3-I (unconjugated form) to LC3-II (lipidated form) is generally regarded as a key regulatory step and indicator of autophagosome formation [32]. In the final stages of autophagy, the autophagosome matures and fuses with the lysosome, where encapsulated cargoes are digested by resident hydrolase activities [9]. Autophagosome maturation and fusion are assisted by several additional regulatory proteins, including small GTPases and lysosome-associated membrane proteins (i.e., LAMP2) [14, 34, 35]. 

**Figure 1:**
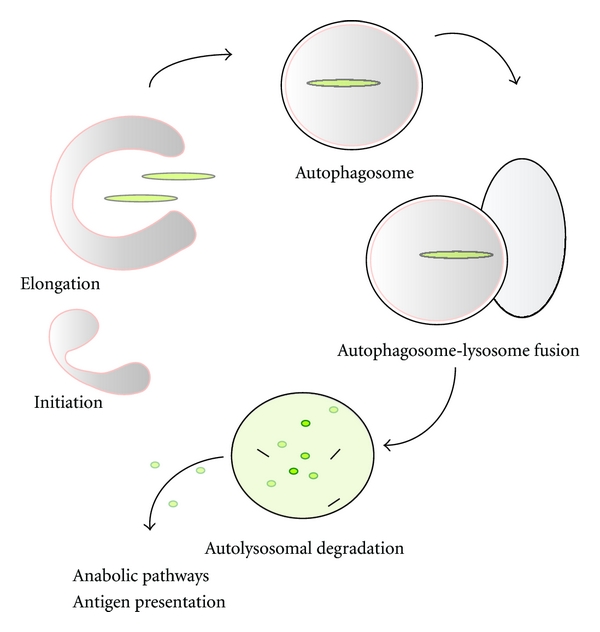
The “macroautophagic” pathway responds to stimulation by various environmental cues including nutrient availability or noxious agents, which result in the accumulation of damaged proteins and/or organelles as well as pathogenic bacteria or viral infection. In the nucleation phase, a preautophagosomal structure develops from subcellular membranes and subsequently evolves into the phagophore or isolation membrane. The isolation membrane then expands to surround and engulf a cytoplasmic “cargo” of material targeted for degradation, culminating in double-membraned autophagosomes. Finally, the fusion of autophagosomes with lysosomes results in the formation of the autolysosome. During the degradative phase of autophagy, the encapsulated contents of autolysosomes are digested by lysosomal degradative enzymes (e.g., cathepsins and other acid hydrolases). The digested contents are then released to the cytosol for reutilization in anabolic pathways.

Recently, additional proteins (i.e., p62^SQSTM1^, NBR1, NDP52, Nix, and others) have been identified in the selection of autophagic cargo. Collectively, these proteins, which are selectively degraded by autophagosomes, act as autophagic adaptors, or cargo receptors for prospective cargos including ubiquitinated proteins, bacteria, or mitochondria [18]. To be considered as an active process, autophagy requires the completion of all steps of the autophagic pathway including substrate turnover, which are collectively referred to as autophagic flux [35]. Changes in LC3B expression and/or conversion, or accumulations of autophagosome numbers, do not necessarily represent active autophagy, as these conditions can arise if the autophagosome-lysosome fusion event and/or subsequent lysosomal processing steps are blocked or impaired [36, 37] (Figure 1). 

## 2. Interaction of Autophagy with Inflammation and Immune Responses

### 2.1. Autophagy and Inflammatory Signaling

The signaling pathways that regulate inflammatory processes now apparently have a role in the regulation of autophagy and *vice-versa*. In addition to classical signals such as starvation and energy exhaustion, several pathogen-associated molecular patterns (PAMPs) have been shown to promote autophagic activation [6]. Recent studies suggest that Toll-like receptors (TLR), the primary cellular sensors for PAMPs, can regulate autophagy through the activation of downstream signaling processes in macrophages and other cells types (reviewed in reference [6]). For example, the TLR9 ligand, bacterial CpG motifs, can induce autophagy in rodent and human tumor cell lines [38]. The screening of TLR ligands for their capacity to induce LC3 puncta (in green fluorescent protein-LC3 assays) revealed that single-stranded RNA (ssRNA) and imiquimod, two model TLR7 ligands, were relatively potent inducers of autophagy [39]. Bacterial LPS, a TLR4 ligand, has been implicated in several studies as a stimulator of autophagic signaling in cultured macrophage cell lines [39, 40]. The ability of LPS to induce autophagy in primary macrophages, however, has been disputed [41]. 

Additional studies suggest that autophagic proteins can modulate responses to viral infections. For example, the IFN-*β* response to double-stranded DNA (dsDNA) is enhanced in mouse embryonic fibroblasts by genetic deletion of Atg9a [42]. For example, the deletion of *Atg5* in macrophages and murine embryo fibroblasts was reported to enhance type 1 interferon production in response to ssRNA virus [43]. The amplification of IFN responses to infection in these cell types was attributed to loss of mitochondrial quality control and enhanced mitochondrial ROS production in response to impaired autophagic processing [43]. In contrast, independent studies reported that the IFN-*α* response to infection with ssRNA virus (i.e., vesicular stomatitis virus) was compromised in *Atg5* ^−/−^ chimeric mice and in *Atg5* ^−/−^ dendritic cells [44]. Independently of autophagy, Atg5 was also shown to play a role in resistance to the intracellular pathogen *Toxoplasma gondi* by facilitating the recruitment of a p47 GTPase to the bacteria containing vacuole [45]. 

Recent observations have revealed a relationship between autophagic proteins and inflammasome-associated proinflammatory cytokine maturation in macrophages [41, 46, 47]. Inflammasomes are cytosolic multiprotein complexes that constitute a novel inflammatory signaling mechanism and which govern the maturation and secretion of select proinflammatory cytokines, such as IL-1*β*, IL-18, and IL-33 [48]. Cytosolic receptors of the NOD-like receptor (NLR) family (i.e., NLRP3 and NLRP1) interact with binding partners to form inflammasome complexes. NLRP3 interacts with an adaptor protein (apoptosis-associated speck like protein containing CARD (ASC)), which recruits and activates the procaspase-1 by proteolytic cleavage [48]. 

Proinflammatory cytokine secretion (IL-1*β* and IL-18) was enhanced in *atg16l1* or *atg7* deleted macrophages in response to LPS [41]. In contrast, *atg16l1* or *atg7* deficiency did not affect TNF and IFN-*β* production or NF-*κ*B pathway activation in macrophages stimulated with LPS [41]. Furthermore,* atg16l1*-deleted mice displayed increased susceptibility to a murine model of colitis, which could be ameliorated by anti-IL-18 therapy [41]. In recent studies, increased activation of IL-1*β* and IL-18 has also been observed in macrophages and monocytes isolated from mice genetically deficient in Beclin 1 and LC3B [46]. 

Cytokine activation in response to LPS and ATP in wild-type macrophages, as well as the amplification observed in LC3B or Beclin 1-deficient macrophages, required the NLRP3 inflammasome pathway [46, 47]. The mechanism by which autophagy deficiency enhanced NLRP3 inflammasome pathway activation was mediated by deregulation of mitochondrial homeostasis, including the enhanced production of mitochondrial ROS and increased mitochondrial membrane permeability transition [46, 47]. The pathway to caspase-1-dependent IL-18 secretion in macrophages was further shown to be blocked by mitochondrial targeting antioxidants [46]. These experiments, taken together, suggest that autophagic proteins dampen inflammasome pathway activation by stabilizing mitochondria and/or maintaining mitochondrial quality control through autophagy. Further research in this area may uncover additional mechanisms. Taken together these studies suggest an important role for autophagic proteins in the dampening of proinflammatory responses, and that warrants further investigation in models of inflammatory disease.

### 2.2. Autophagy and Adaptive Immunity

**Figure 2:**
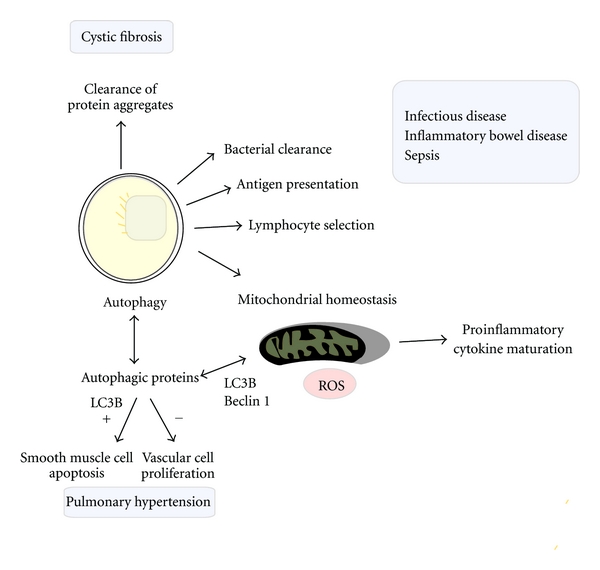
Autophagy as an adaptive cellular process potentially impacts the progression of inflammatory diseases by several possible mechanisms. (i) Autophagy, by acting as a “xenophagic” response, directly participates in bacterial clearance, through the encapsulation and lysosomal delivery of invading bacteria for degradation. (ii) Autophagic processes can assist in antigen presentation through the digestion of invading pathogens. (iii) Autophagic proteins play a role in the dampening proinflammatory responses, including proinflammatory cytokine secretion, through the maintenance of mitochondrial quality. (iv) Autophagic degradation of denatured protein aggregates may play a protective role in tissues such that impaired function has been associated with diseases such as cystic fibrosis [5]. (v) Autophagic protein LC3B potentially regulates other cellular processes as recently described in a model of hypoxia-induced pulmonary hypertension. In these studies, LC3B was found to inhibit vascular cell proliferation and promote smooth muscle cell apoptosis, collectively associated with protection in this model [29].

Autophagy plays critical role in bacterial clearance mediated by autophagosomal sequestration and subsequent autolysosome-dependent degradation and in the regulation of the cytokine response [5] (Figure 2). In addition to these roles, recent studies also indicate that autophagy can participate in adaptive immune responses, including antigen presentation, and in the maintenance of lymphocyte function [5, 49]. The discovery that autophagosomes can fuse with and transfer content to major histocompatibility complex (MHC) Class II loading compartments illustrates the importance of this relationship [50]. The immune system detects pathogen-derived antigens (i.e., peptides) and initiates a response through MHC Class I and II loading compartments. The peptide fragments generated by intracellular degradation of bacteria, including autolysosomal degradation, are displayed on MHC Class I and II molecules. Class I MHC molecules are generally present in most cell types and assist in antigen presentation to CD8^+^ T cells. Inhibition of autophagy by chemical inhibitors or genetic knockdown of select autophagic proteins (i.e., Atg5) typically does not affect MHC Class I antigen presentation [49]. However, autophagy induction in target cells was shown to increase their ability to act as immunogens for dendritic cell cross-presentation to CD8^+^ T cells [51]. Furthermore, autophagy induced during HSV-1 infection enhances the presentation of viral-derived antigen on MHC Class I molecules [52]. Class II MHC molecules (which are found specifically in antigen-presenting cells such as macrophages, B cells, and dendritic cells) present bacterial fragments to CD4^+^ T cells, which mediate immune responses from other cell types. Genetic interference of Atg12 was shown to inhibit MHC Class II antigen presentation to CD4^+^ T cells during Epstein-Barr virus infection [53]. Genetic deletion of *Atg5 *also suppressed the processing and presentation of herpes simplex virus-2- (HSV-2-) derived antigen on MHC Class II molecules and enhanced vulnerability of mice to HSV-2 infection [54]. Recent studies have identified a novel role for autophagy in the generation of a self-tolerant T cell repertoire. Constitutively, highly expressed autophagy in thymic epithelial cells delivers endogenous proteins to MHC Class II molecules and contributes to CD4^+^ T cell selection. The grafting of embryonic thymi from *Atg5* ^−/−^ mice into athymic nude mice was shown to promote systemic lymphoid infiltration [55]. Taken together, these examples suggest that autophagy and/or autophagic proteins play multivariate roles in immune system function. 

## 3. Autophagy in Inflammatory Diseases

### 3.1. Autophagy in Crohn's Disease

Crohn's disease is a chronic inflammatory bowel disease characterized by inflammation, ulceration, and neutrophil influx in the intestinal epithelia. The pathophysiological mechanisms of Crohn's disease remain unclear but may involve excess inflammatory responses, abnormal Paneth cell granule secretion, and impaired intracellular bacterial clearance [56]. Recent human studies have suggested links between autophagy and Crohn's diseases. Genome-wide association studies (GWAS) have revealed small nucleotide polymorphisms (SNPs) in autophagy genes such as ATG16lL and in additional genes now known to influence autophagic processing (i.e., NOD2 and IRGM) associated with susceptibility to Crohn's disease [57–61]. 

The first of these to be described, a T300A variant in the ATG16lL gene, has been identified as an associated risk factor for Crohn's disease. ATG16lL plays a key role in autophagosome formation [57, 58]. Genetic deletion of ATG16lL impairs autophagosome formation and autophagic processing of protein and, furthermore, promotes IL-1*β* production in macrophages in response to LPS stimulation [41]. The mechanism by which the T300A mutation in ATG16lL compromises autophagic function remains unclear [6]. 

Variants in the gene encoding immunity-related p47 guanosine triphosphatase (IRGM) were associated with Crohn's disease in a recent GWAS [62]. Its murine homologue Irgm1 can regulate intracellular autophagy in response to IFN-*γ* stimulation and starvation. Mice rendered deficient in *Irgm1* displayed an impaired ability to clear intracellular bacteria [63]. Recent studies suggest that human IRGM regulates autophagy through dynamic interactions with mitochondria [64, 65]. IRGM associates with mitochondria by binding to the phospholipid cardiolipin, a constituent of the mitochondrial inner membrane, and consequently promotes mitochondrial membrane depolarization and mitochondrial fission [65]. 

At least three mutations in the gene encoding nucleotide-binding oligomerization domain 2 (NOD2), also known as CARD15, including small nucleotide polymorphisms (R702W and G908R) and a frameshift mutation (L1007fsinsC) have been found in association with Crohn's disease [66–73]. NOD2, a protein of the NLR family, functions as an intracellular bacteria sensor. NOD2 activates signaling pathways in response to stimulation with bacteria-derived peptides [74]. Muramyl dipeptide (MDP), a component of the bacterial peptidoglycan cell wall, induces autophagy in intestinal epithelial cells, and thereby also promotes autophagy-dependent bacterial clearance [75]. The induction of autophagy by MDP requires NOD2 and ATG16L and involves physical interaction of NOD2 with ATG16L [76]. NOD2 recruits ATG16L to the membrane site of bacterial entry and facilitates association of LC3B with bacteria [76]. The expression of NOD2 genetic variants associated with Crohn's disease results in impaired autophagic processing of pathogens (i.e., *Salmonella typhimurium*) by epithelial cells in response to treatment with NOD2 ligand [75]. In addition to stimulation of autophagy-dependent bacterial clearance, NOD2 was recently shown to also regulate dendritic cell MHC Class 2 dependent antigen presentation to CD4^+^ T-cells [77]. Recent studies also show that inhibition of the autophagy process or expression of the ATG16lL T300A variant leads to increased proinflammatory cytokine responses (i.e., IL-1*β* and IL-6) in human primary immune cells in response to stimulation with NOD2 ligands [78]. 

Taken together, defects of autophagic activity as the result of mutations in autophagy-associated genes (i.e., *ATG16L1 and IRGM*) and bacterial sensors (NOD2) have been associated with the impaired clearance of harmful bacterial species associated with Crohn's disease, impaired antigen presentation, and also with the higher production of proinflammatory cytokines implicated in the pathogenesis of Crohn's disease. 

### 3.2. Autophagy in Respiratory Infections

Autophagy can exert antibacterial and antipathogen functions, which were originally demonstrated in several infectious disease models using live bacteria [79, 80]. The general role of autophagy in host defense against various microbes including bacteria, viruses, and parasites has now been widely recognized [6]. 

Phagocytosis of nonpathogenic mycobacteria by macrophages leads to autophagy and apoptosis, which results in the termination of the microbe. However, phagocytosis of pathogenic mycobacteria inhibits the autophagy pathway with the acidification of phagosomes and lysosomal fusion [81]. Mutations in *NOD2,* a pathogen-recognition receptor critical for bacterial autophagy [76, 77], are associated with vulnerability to microbial infection with the etiologic agent of leprosy, *Mycobacterium leprae *[82].

In the case of *M. tuberculosis*, the mycobacteria remains and replicates in immature phagosomes. In addition, instead of stimulating macrophage apoptosis, phagocytosis of *M. tuberculosis* promotes necrotic cell death, which promotes bacteria dispersal to uninfected cells. As a result, reduced mycobacterial antigen presentation and chronic *M. tuberculosis* infection occur [83]. However, experimental stimulation of autophagy can reduce intracellular replication and survival of *M. tuberculosis *[79, 83–85]. Conversely, chemical inhibitors of autophagy promote infection [80, 86].

IFN-*γ* production makes an important contribution to host defense against *M. tuberculosis. *Macrophages stimulated with IFN-*γ* induce autophagy, and this response facilitates the resolution of infection [80, 87]. IFN-*γ*-stimulation can thereby bypass the inhibition of lysosomal fusion of virus containing phagosomes, leading to the destruction of the organism by p62^SQSTM1^-dependent selective autophagy [87], and resolution of infection. IFN-*γ* induced autophagy involves the p47 guanosine triphosphatase IRGM-1 [64, 65, 80, 88]. Interestingly, small nucleotide polymorphisms occurring in the IRGM-1 gene, as implicated in Crohn's disease, were recently also linked to increased susceptibility to *M. tuberculosis* infection [89, 90]. Finally, autophagic process may assist in the generation of antivirulence factors against this organism through degradation of substrate proteins [87, 91].

Given that *M. tuberculosis* is the pathogenic agent causing tuberculosis, this bacterium is a major contributor to global disease burden [92]. Therefore, therapeutic strategies involving autophagy pathway manipulation to reduce infection and promote adaptive immunity to this organism, and to other related pathogens, may be of considerable interest. Additional studies implicate autophagy in defense against other respiratory pathogens, such as *Legionella pneumophila*, the causative agent in Legionnaire's disease. For example, genetic deficiency of *atg9 *was shown to promote the growth of *Legionella pneumophila*, which suggests a role of autophagy in defense against this organism [93, 94]. 

Recent studies have shown that the fundamental mechanisms of host pathogenic response are conserved in lower eukaryotes [95–97]. Also, autophagy remains critical for normal cellular development of the social amoeba in *Dictyostelium discoideum* [98]. Therefore, *D. discoideum* has become a widely used model system for the study of bacterial infection and autophagy [99–101]. Infection of *D. discoideum* with *Legionella pneumophila* causes a large increase in differentially regulated autophagy-related genes, including ATG8, ATG9, and ATG16 [102]. However, recent studies have shown that *L. pneumophila* can undergo replication in autophagy mutants of *D. discoideum *[103].

### 3.3. Autophagy in Sepsis

Sepsis remains a leading cause of mortality in intensive care units. This condition arises as a consequence of systemic responses to inflammation caused by acquired bacterial, fungal, parasitic, or viral infections and may lead to multiple organ failure [104]. 

To date, little is known of the role of autophagy in sepsis. Marked autophagosome accumulation has been observed in the livers of patients who die from sepsis [105]. However, it currently remains unclear whether this observation represents increased autophagic activity (flux) in sepsis patients or inhibition of autophagic processing which leads to the inappropriate accumulation of autophagosomes. Genetic deletion of critical autophagic proteins has recently been shown to increase sepsis-induced inflammatory responses in mice subjected to the cecal-ligation and puncture (CLP) model of polymicrobial sepsis [46]. Similar results were observed in mice challenged with LPS injection [46]. Furthermore, *BECN1* ^+/−^ mice and *LC3B* ^−/−^ mice were found to be susceptible to the lethal effects septic shock in mice, and to express higher levels of IL-18, one of the inflammasome-associated cytokines in the plasma [46]. Collectively, these studies suggest a potential link between autophagy and inflammatory responses during the pathogenesis of sepsis.

## 4. Autophagy in Pulmonary Disease

Recent evidence from this laboratory and other, suggests that autophagy may be critically involved in other noninfectious pulmonary diseases, where inflammation has been implicated. In these cases, additional functional aspects of autophagy may indirectly affect inflammation by limiting tissue injury. In two illustrative examples, we describe recent work illustrating how autophagy may impact the pathogenesis of pulmonary hypertension and cystic fibrosis. 

### 4.1. Role of Autophagic Protein LC3B in Pulmonary Hypertension

Recent studies from this laboratory have sought to determine the involvement of autophagic proteins in pulmonary arterial hypertension (PAH). PAH is a complex disease of varying etiologies which include idiopathic and forms as well as other subtypes (i.e., associated with left heart disease, HIV infection, etc.). PAH is characterized mainly by vasoconstriction, increased pulmonary artery pressure, thickening and fibrosis of the artery, which may lead to cardiac dysfunction, and right ventricular hypertrophy [106, 107]. We examined the prospective role of autophagic proteins in an experimental mouse model of chronic hypoxia-induced pulmonary hypertension. Exposure to chronic hypoxia in mice resulted in the increased expression of LC3B and its conversion of LC3B-II in the lung. Increased LC3B staining was also observed in small pulmonary vessels of animals subjected to hypoxia. Moreover, hypoxic lungs contained elevated numbers of autophagosomes, as detected by electron microscopy. Importantly, mice genetically deleted for LC3B (*LC3B* ^−/−^) displayed increased indices of pulmonary hypertension, including increased right ventricular systolic pressure, and Fulton's Index relative to wild-type mice, after chronic hypoxia [29]. These results identified an endogenous role for autophagic protein LC3B in the regulation of protective processes during the development of pulmonary hypertension. Genetic deletion of LC3B aggravated the hypertensive phenotype as the result of hypoxia exposure. These observations were corroborated with observations *in vitro* of increased vascular cell (i.e., endothelial and smooth muscle) proliferation and impaired smooth muscle cell apoptosis in after LC3B-specific genetic knockdown. These experiments, which have used LC3B knockout or knockdown strategies, suggest a specific role for the autophagic protein LC3B, in vascular responses to hypoxia, and associated pathogenic processes implicated in the development of pulmonary hypertension. However, these experiments did not unequivocally establish a specific role for autophagic activity in these phenotypes. The authors could not exclude that LC3B exerts effects on signaling processes independently of the process of autophagy. In contrast, the vascular changes recorded in *Beclin 1^+/−^* mice were not statistically significant. Experiments using additional autophagy protein knockout mice (i.e., Atg5) may be warranted.

The relevance of these findings to clinical disease, was supported by similar observations in human tissues from patients with pulmonary hypertension. Human lung tissue isolated from patients with pulmonary hypertension (PH) of various etiologies, including PAH, displayed increases in the total expression of LC3B, and in the levels of its activated (PE-conjugated) form LC3B-II, when compared to lung tissue from patients free of pulmonary vascular disease. The expression of LC3B was markedly increased in the endothelial cell layer, as well as in the adventitial and medial regions of large and small pulmonary resistance vessels from PH lung, relative to normal vascular tissue. These results, taken together, suggest that autophagic proteins may potentially be exploited for the prevention and/or treatment of vascular disease in humans [29]. 

### 4.2. Autophagy in Cystic Fibrosis: A Role for “Aggrephagy”

In addition to effects on bacterial clearance and the resolution of inflammation, autophagy may exert additional functions that could ameliorate inflammatory diseases. One of these functions is the selective clearance of aggregated and denatured protein, a process termed “*aggrephagy*”. This function is exemplified by recent studies on the role of autophagy in cystic fibrosis (CF), a debilitating autosomal recessive disorder. CF patients are marked by the collection of misfolded proteins in the airway epithelia due to mutations (ΔF508 and others) in the gene encoding the cystic fibrosis transmembrane conductance regulator (CFTR) [1]. 

The pathological features of CF include aberrant accumulation of hyperviscous mucous in the airways, impaired mucociliary clearance, and increased inflammation. Lung injury may also arise from secondary infections (i.e., *Staphylococcus aureus, Pseudomonas aeruginosa, *etc.) [108]. 

Recent studies demonstrate that human airway epithelial cells from CF patients, which bear the mutation in the CFTR gene, have an impaired autophagic response. In response to starvation, a classical inducer of autophagy, these cells exhibited reduced autophagosome formation and accumulation of p62^SQSTM1^. The human epithelial cells with CFTR mutation also displayed an abnormal accumulation of polyubiquitinated protein aggregates indicative of impaired aggresome clearance. In normal epithelial cells, mutation and/or loss of function of CFTR was associated with elevated reactive oxygen species (ROS) production and increased tissue transglutaminase 2 (TG2) levels, an important factor of inflammatory response in CF. Activation of these pathways caused a loss of function in Beclin 1 and the Beclin 1/PIK3C3 complex and, as a result, loss of autophagic function. Beclin 1 overexpression, or the application of cystamine or other antioxidants restored Beclin 1 function and autophagy and reverted the CF airway phenotype in human CF nasal biopsies*, in vivo *in *Scnn1b*-transgenic mice (a model of CF), and *Cftr*F508del homozygous mice as well as in cells expressing mutant CFTR (ΔF508) *in vitro*. Reconstitution of Beclin 1 levels also restored the membrane trafficking of mutant CFTR and reduced its accumulation in aggresomes. Furthermore, recent studies have shown that the accumulation of p62^SQSTM1^, an LC3-binding and ubiquitin-binding protein, in the context of impaired autophagy, promotes the aberrant accumulation of intracellular protein aggregates in human CF airway epithelial IB3-1 cells. Thus, defective CFTR causes impaired autophagy processing, which favors the accumulation of aggresomes, and lung inflammation [5, 109]. In conclusion, selective targeting the autophagic pathway may be included in the design of therapeutics for the treatment of CF. 

## 5. Final Remarks

Current studies indicate that autophagic processes can exert a significant impact on the regulation of inflammation, on the resolution of infection, and on immune responses to invading pathogens. These observations collectively implicate autophagy as an important modulator of disease pathogenesis. The bacterial clearing function of autophagy may contribute to host defenses in diseases involving bacteria, such as sepsis, inflammatory diseases of the bowel, and respiratory infections. Furthermore, autophagy may serve a function in downregulating proinflammatory cytokine production implicated in tissue injury, which may also exert a protective role in inflammatory diseases not necessarily involving bacterial infection. The ability of autophagy to clear aggregated protein (i.e., aggrephagy) as well as to maintain mitochondrial homeostasis (i.e., mitophagy) may also play supporting roles in protection against diseases associated with inflammation. Finally, the possibility remains that autophagic proteins may regulate cellular processes independently of their role in regulating autophagic activation. Much progress has accumulated in understanding these relationships in select infectious and inflammatory diseases. Further research will determine whether the autophagic pathway can be manipulated for therapeutic gain in the treatment of inflammatory diseases and/or other diseases of the lung and cardiovascular system [110, 111]. 

## Abbreviations

ATG: Autophagy-related gene
CF: Cystic fibrosis
CFTR: Cystic fibrosis transmembrane conductance regulator
CLP: Cecal ligation and puncture
GWAS: Genome-wide association study
IFN: Interferon
IRGM: Immunity-related p47 guanosine triphosphatase
LC3B: Microtubule-associated protein-1 light chain 3
LPS: Lipopolysaccharide
mTOR: Mammalian target of rapamycin
mTORC1: mTOR complex 1
MHC: Major histocompatibility complex
NLR: NOD-like receptor
NLRP3: NOD-like receptor protein-3
NOD2: Nucleotide-binding oligomerization domain 2
p62^SQSTM1^: 62-kDa protein, selective autophagy substrate
PAH: Pulmonary arterial hypertension
PH: Pulmonary hypertension
PIK3C3: Phosphatydylinositol-3-kinase (Class 3)
PMN: Polymorphonuclear cells
ROS: Reactive oxygen species
SNP: Small nucleotide polymorphism
ssRNA: Single stranded RNA
TLR: Toll-like receptor
ULK-1: Uncoordinated-51-like protein kinase.
